# Prickly ash seeds can promote healthy production of sheep by regulating the rumen microbial community

**DOI:** 10.3389/fmicb.2024.1364517

**Published:** 2024-05-20

**Authors:** Dengpan Li, Qiao Li, Xueyi Ma, Huihui Wang, Chunhui Wang, Haoyu Wang, Zhanjing Liu, Taotao Li, Youji Ma

**Affiliations:** ^1^College of Animal Science and Technology, Gansu Agricultural University, Lanzhou, China; ^2^Gansu Key Laboratory of Animal Generational Physiology and Reproductive Regulation, Lanzhou, China; ^3^Tianzhu County Animal Disease Prevention and Control Center, Wuwei, China

**Keywords:** prickly ash seeds, 16S rRNA, rumen microbial, sheep, production property

## Abstract

This study aimed to investigate the effect of prickly ash seeds (PAS) on the microbial community found in rumen microbes of Hu sheep by adding different percentages of prickly ash seeds and to carry out research on the relation between rumen flora and production performance. Twenty-seven male lambs of Hu sheep were classified into three groups based on the content of prickly ash seeds (PAS) fed for 90 days, i.e., 0%, 3%, and 6%. At the end of the feeding trial, rumen fluid samples were collected from six sheep in each group for 16S amplicon sequencing. The results showed that the addition of prickly ash seeds significantly increased both Chao1 and ACE indices (*P* < 0.05), and the differences between groups were greater than those within groups. The relative content of *Bacteriodota* decreased, and the relative content of *Fusobacteriota, Proteobacteria, Acidobacteriota*, and *Euryarchaeota* increased. The relative content of *Papillibacter* and *Saccharofermentans* was increased at the genus level, and the relative content of *Bacteroides* and *Ruminococcus* was decreased. The test group given 3% of prickly ash seeds was superior to the test group given 6% of prickly ash seeds. In addition, the addition of 3% of prickly ash seeds improved the metabolism or immunity of sheep. *Fusobacteriota* and *Acidobacteriota* were positively correlated with total weight, dressing percentage, and average daily gain (ADG) and negatively correlated with average daily feed intake (ADFI), feed-to-gain ratio (F/G), and lightness (L^*^). *Methanobrevibacter* and *Saccharofermentans* were positively correlated with ADG and negatively correlated with ADFI and L^*^. In conclusion, under the present experimental conditions, the addition of prickly ash seeds increased the abundance and diversity of rumen microorganisms in Hu sheep and changed the relative abundance of some genera. However, the addition of 6% prickly ash seeds may negatively affect the digestive and immune functions in sheep rumen.

## 1 Introduction

Complex and large microbial communities inhabit the rumen of ruminants and play a key role in ruminant health, growth performance, and immunity (Malmuthuge and Guan, 2017; Schären et al., 2018). Microorganisms in the rumen include four major groups: protozoa, fungi, archaea, and bacteria. Among the bacteria, *Firmicutes* and *Bacteroidetes* are the most abundant. Most of the species in *Firmicutes* are associated with carbohydrate metabolism (Fernando et al., 2010; Hart et al., 2018), whereas *Bacteroidetes* are mainly associated with starch digestion and metabolism (Jami and Mizrahi, 2012; Snelling and Wallace, 2017). In addition, there are other groups of bacteria such as *Fibrobacterota, Gracilibacteria, Spirochaetota*, and *Euryarchaeota* that work together to maintain and mutually regulate the stability and balance of the rumen's internal environment. It has been found that the composition of the microbial community in the rumen can be affected by various factors such as diet, environmental factors, species, sex, and age, of which diet composition, nutritional level, and feed intake are considered to be the key factors affecting the rumen microbial community. Henderson et al. measured rumen microbial samples from 35 countries and 32 animal species to assess whether they were influenced by diet, host, or geographic location and found that differences in microbial community composition were primarily attributable to diet, with a lesser influence of the host (Henderson et al., 2015). In addition, in long-term feeding trials, it was found that adding enzyme preparations, plant extracts, probiotics, herbal additives, and other substances to animal feed can improve the gastrointestinal health of livestock and poultry; enhance the body's immune system; promote the digestion and absorption of nutrients, which will have a positive impact on the growth of livestock and poultry; and reduce the cost of feeding to a certain extent (Mao et al., 2023; Wang et al., 2023a).

Prickly ash is a small perennial deciduous tree of the family Rutaceae. It has high economic value in terms of food and medicinal use and has become an important source of economy for farmers in some areas. Prickly ash seeds are the main byproduct of prickly ash production, accounting for approximately 60% of the total weight of prickly ash. Prickly ash seeds can be processed into prickly ash oil (an ideal edible vegetable oil) and can also be used as herbal medicine. Long-term studies have found that prickly ash seeds are rich in dietary fiber, melanin, polyphenols, flavonoids, quercetin, volatile oils, and other natural substances (Tang et al., 2014; Yang et al., 2014), which have better anti-inflammatory and antibacterial (Hou et al., 2021), antioxidant, antitumor, cholesterol-lowering, and immune-enhancing properties (Fortuoso et al., 2019; Zhong et al., 2022). In addition, the antimicrobial peptides of prickly ash seeds have antimicrobial activity, inhibiting *Bacillus subtilis, Escherichia coli, Salmonella, and Staphylococcus aureus* (Hou et al., 2019). Prickly ash seeds inhibit lung inflammation and tissue damage during asthma (Wang et al., 2016; Phuyal et al., 2020). Prickly ash seed oil extracted from prickly ash seeds inhibits osteoclastogenesis and anti-malignant melanoma (Pang et al., 2019; Zhang et al., 2022a). In recent years, a number of scholars have carried out studies using prickly ash seeds and their derivatives in animals. For example, the addition of prickly ash seeds to diets has the potential to enhance pork quality and growth performance (Song et al., 2020). Prickly ash essential oil can promote nutrient digestion and absorption as well as increase intestinal health in sheep, while a portion of the rumen microbiota was significantly associated with differential metabolite and enzyme activities (Zhang et al., 2022a,b).

With the development of modernization and large-scale farming, the concept of green and healthy farming has been proposed in animal husbandry. In addition, prickly ash seeds have the advantages of naturalness, non-resistance, and multifunctionality, making it an ideal green feed. As the raw materials are abundant, they do not need any processing, resulting in low economic cost. Previous experiments have shown that including prickly ash seeds in the diet can improve the beneficial bacteria in the intestinal tract of Hu sheep lambs, reduce harmful bacteria, reduce inflammation, and increase the immunity of sheep (Li et al., 2023a). Therefore, 16s rRNA sequencing technology was used in this study to compare the bacterial community structure and distribution in the rumen of sheep supplemented with 0%, 3%, and 6% prickly ash seeds and to analyze the correlation between rumen microorganisms and production traits. This study aimed to augment the limitation in studies on the effects of prickly ash seeds on the ruminal bacterial community and to find the key microorganisms affecting the production performance of sheep through a study on the correlation between rumen microbes and production traits so as to provide beneficial help for promoting the healthy growth of ruminants.

## 2 Materials and methods

### 2.1 Ethics statement

All experimental designs and procedures were approved by the Animal Care Committee for Animal Care and Experimental Procedures established by the Ministry of Science and Technology of the People's Republic of China (Approval No. 2006-398).

### 2.2 Animals and experimental design

Twenty-seven healthy 3-month-old Hu male sheep lambs (aged 90 ± 5 days; 25.66 ± 3.03 kg body weight) were selected from June to September 2022 at Dongwu Good Breeding Sheep Breeding Base, Dongxiang County (Linxia Prefecture, Gansu Province, China). They were randomly divided into three groups of nine lambs each. The three groups were supplemented with 0% (CK group), 3% (A group), and 6% (B group) of prickly ash seeds in the basal diet. At the end of the fattening test, six sheep in each group were randomly selected to determine production traits after slaughter, and rumen fluid was collected for 16S rRNA sequencing and analyzed for the correlation between the diversity of rumen flora and production traits.

### 2.3 Test diet

The basal diet was formulated with reference to the Nutritional Requirements of Chinese Meat Sheep and made into a total mixed ration (TMR). The composition and nutrient content of the experimental basal diet are shown in Table 1. Prickly ash seeds were purchased from the grain market in Guanghe County, and their conventional nutrient content (Supplementary Table S1) is given in Li et al. (2023a).

**Table 1:** Composition and nutrient levels of basal diet (dry matter basis).

**Diet ingredient**	**CK**	**A**	**B**
Ingredients DM %			
Corn	29	29	29
Wheat bran	12	12	12
Soybean meal	14	14	14
Rapeseed meal	4	4	4
Prickly ash seeds	0	3	6
Alfalfa hay	13	10	10
Wheat straw hood	10.3	10.3	10.3
Whole corn silage	14	14	11
Premix	1	1	1
NaCl	1	1	1
Limestone	1	1	1
CaHCO_3_	0.7	0.7	0.7
Total	100	100	100
Nutrient levels			
Metabolic energy (MJ/kg)	9.75	9.76	9.76
Crude protein (%)	15.38	15.35	15.59
Crude fat (%)	2.50	3.13	3.68
NDF (%)	28.35	28.22	28.18
ADF (%)	16.27	16.20	16.17
Ca (%)	0.97	0.93	0.93
P (%)	0.52	0.53	0.54

DM, dry matter; NDF, neutral detergent fiber; ADF, acid detergent fiber. The premix provided the flowing per kg of diets: Fe 75 mg, Cu 14 mg, Zn 80 mg, Se 0.25mg, I 0.25 mg, Co 0.3 mg, Mn 400 mg, VA 3000 IU, VD 500 IU, and VE 200 IU. ME was a calculated value, while the others were measured values.

### 2.4 Feeding management

Before the start of the trial, the pen was thoroughly disinfected, the sheep were ear-tagged and vaccinated according to the procedure, and then each sheep was weighed and placed in pens. At the beginning of the trial, feeding was carried out at 07:00 am and 18:00 pm every day, during which the lambs had *ad libitum* access to food and drink, and the pens were disinfected regularly.

### 2.5 Sample collection and processing

At the end of the fattening test, six test sheep with close body condition were randomly selected from each group. The sheep were slaughtered, and the rumen fluid was collected after 12 h of fasting and finally filtered through a gauze into 50-mL centrifuge tubes. The centrifuge tubes were immediately placed in a liquid nitrogen tank and brought back to the laboratory, and there, they were transferred to a −80°C refrigerator for storage for the determination of rumen microbiota diversity.

### 2.6 16S rRNA amplicon sequencing

#### 2.6.1 DNA extraction

Total microbial DNA was extracted from rumen samples with reference to the requirements of the TGuide S96 Magnetic Bead Method Soil/Fecal Genomic DNA Extraction Kit [Tiangen Biotechnology Co., Ltd. (Beijing, China)]. The concentration of the DNA samples was determined by an enzyme marker and then qualitatively detected by 2% agarose gel electrophoresis. The DNA samples that met the requirements for online library construction were sent to Wuhan Metavir Biotechnology Co. (Wuhan, China) for 16S amplicon sequencing.

#### 2.6.2 Library construction and on-line sequencing

Using DNA diluted in sterile water as the template, specific primers were synthesized with barcode sequences (338F: ACTCCTACGGGGAGGCAGCAG; 806R: GGACTACHVGGGGTWTCTAAT) according to the sequence of primers in the V3–V4 region for PCR amplification. All PCR reactions were carried out with 15 μL of Phusion^®^ High-Fidelity PCR Master Mix [New England Biolabs (Beijing, China) Ltd.], with 0.2 μM of forward and reverse primers, and approximately 10 ng template DNA. Thermal cycling consisted of initial denaturation at 98°C for 1 min, followed by 30 cycles of denaturation at 98°C for 10 s, annealing at 50°C for 30 s, and elongation at 72°C for 30 s, and final denaturation at 72°C for 5 min. Sequencing libraries were generated using the TruSeq^®^ DNA PCR-Free Sample Preparation Kit [Illumina (Shanghai, China) Ltd.] following the manufacturer's instructions, and index codes were added. The library quality was assessed on the Qubit@ 2.0 Fluorometer [Thermo Scientific (Shanghai, China) Ltd.] and Agilent Bioanalyzer 2,100 system (Beijing, China). Finally, the library was sequenced on an Illumina NovaSeq 6,000 platform (Shanghai, China), and 250-bp paired-end reads were generated.

#### 2.6.3 Sequencing data processing

The downstream data (raw PE) from Illumina NovaSeq sequencing were spliced and quality-controlled to obtain clean tags, and then, chimera filtering was performed to obtain effective tags that could be used for subsequent analyses. The UPARSE algorithm (USEARCH v7, http://www.drive5.com/uparse/) was used to cluster the effective tags of all the samples into operational taxonomic units (OTUs) according to the 97% sequence similarity principle. The sequences of OTUs were then annotated with the SSUrRNA database of SILVA138.1 (http://www.arb-silva.de/) using the mothur method to obtain the microbial community composition of each sample or subgroup at each subgroup level (set the threshold to 0.8 ~ 1). Rapid multiple sequence comparisons were performed using MAFFT (v7.490, https://mafft.cbrc.jp/alignment/software/) software, and then, the data were homogenized across samples. The alpha-diversity and beta-diversity analyses were performed. Qiime software (Version 1.9.1) was used to calculate Observed_otus, Chao1, Shannon, Simpson, ace, Goods-coverage, and PD_whole_tree indices. Rarefaction curve, rank abundance curves, and species accumulation curves were plotted using R software (Version 4.1.2), and differences between groups were analyzed using R software for the alpha-diversity index. QIIME software (Version 1.9.1) was used to calculate the UniFrac distance and construct the UPGMA clustering tree. Principal component analysis (PCA), principal coordinates analysis (PCoA), and Non-Metric Multi-Dimensional Scaling (NMDS) plots were drawn using R software (Version 4.1.2). Analysis of similarity (ANOSIM) uses the anosim function of the R vegan package. Linear discriminant analysis effect size (LEfSe) analyses were performed using LEfSe software with the default filter value of LDA score set to 4. Finally, the 16S rRNA gene sequences were analyzed for functional prediction in Kyoto Encyclopedia of Genes and Genomes (KEGG) and Cluster of orthologous group (COG) databases using PICRUSt2. Other graphics used OmicStudio (https://www.omicstudio.cn/online).

### 2.7 Statistical analysis

The experimental data were preliminarily sorted out by Excel 2021 software, and then, the one-way ANOVA process of SPSS 26.0 statistical software was used to conduct the one-way ANOVA. Duncan's method was used to conduct multiple comparisons test, and the results were represented by mean ± standard deviation. A *p*-value of < 0.05 indicates statistically significant difference, and 0.05 ≤ *P* < 0.1 indicates that the difference tends to be significant. With reference to the previous results on production traits by our research group (Ma et al., 2024), we used Spearman's correlation test to analyze the correlation between rumen microorganisms and production traits. The greater the correlation coefficient, the stronger the correlation between the variables. Spearman's correlation coefficient greater than 0.5 means that there is a strong positive correlation between the two variables, and a correlation coefficient < 0.5 means that there is a strong negative correlation between the two variables.

## 3 Results

### 3.1 Sequencing results and bacterial diversity

**Figure 1:**
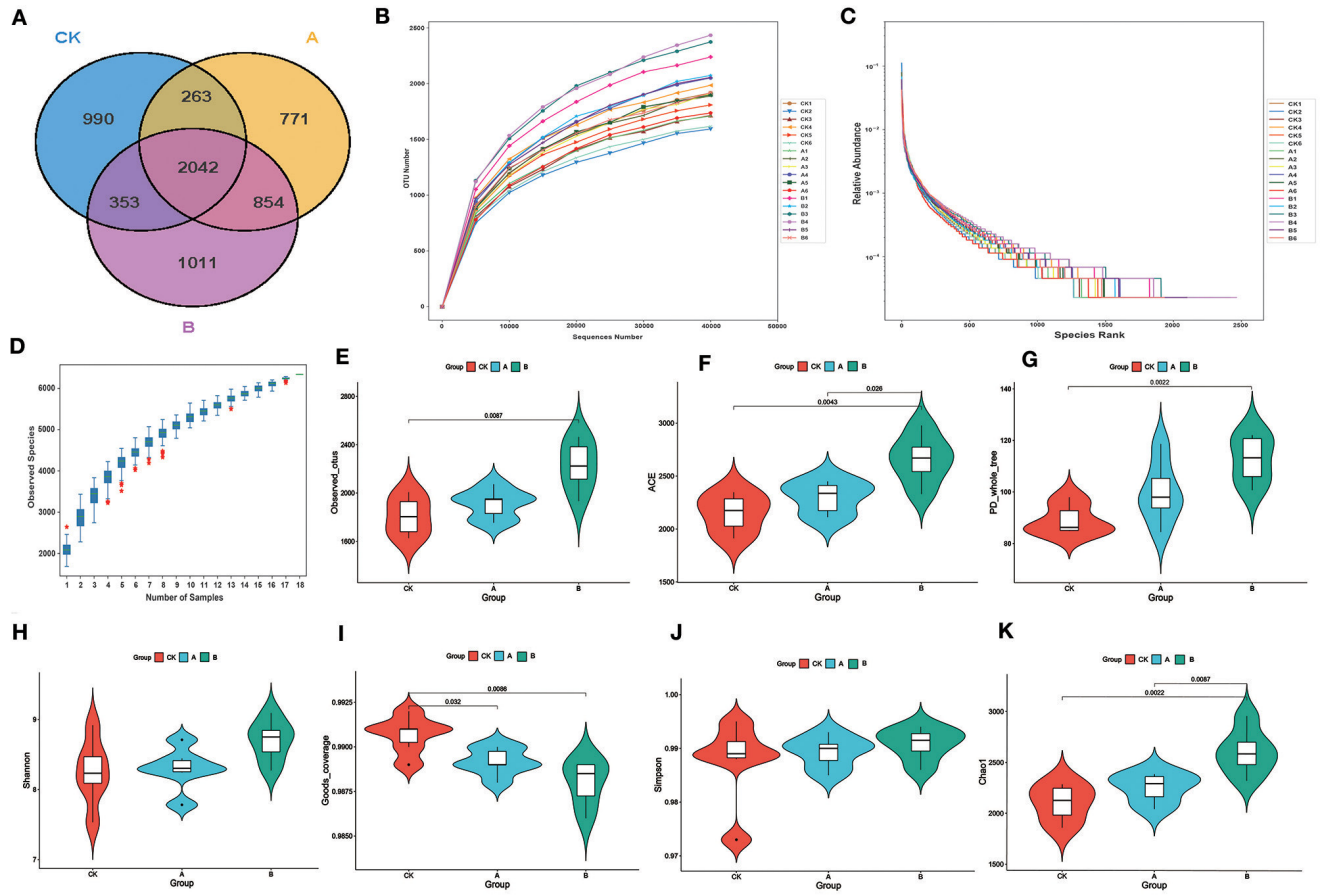
Sequencing results and statistical analysis of diversity. **(A)** The Venn diagram shows three groups of common or unique OTUs. **(B)** Rarefaction curve for all samples. **(C)** Rank abundance. **(D)** Species accumulation boxplot. **(E)** Violin plots of the number of observed_otus values between the three groups. **(F)** Violin plots of the number of ACE index values between the three groups. **(G)** Violin plots of the number of PD_whole_tree index values between the three groups. **(H)** Violin plots of the number of Shannon index values between the three groups. **(I)** Violin plots of the number of Goods_coverage between the three groups. **(J)** Violin plots of the number of Simpson index values between the three groups. **(K)** Violin plots of the number of Chao1 index values between the three groups.

A total of 1,379,355 reads pairs were obtained by 16S amplicon sequencing. A total of 1,340,281 clean tags were generated after paired-end reads were spliced and filtered, and 1,047,982 effective tags produced by chimeras were removed. At least 56,078 clean tags were generated for each sample, and 74,460 clean tags were generated on average. The average content of Q30 was 94.07%, and the average content of GC was 54.15%. On the principle of dividing different OTUs based on a sequence similarity >97%, there were 3,648, 3,930 and 4,260 OTUs in groups CK, A, and B, respectively (Figure 1A), with the lowest number of OTUs unique to group CK and the highest number of OTUs in group B. The number of OTUs common to each group was 2,042. The unique OTUs in each group were 990, 771, and 1,011, respectively. The sample sequencing rarefaction curve show that the curves flatten out as the number of samples sequenced increases, indicating an adequate amount of data (Figure 1B). Species accumulation box plots were used as a judgement of the adequacy of the sample size. The absence of a sharp increase in the position of the box plots indicated that the sampling was adequate and that data analysis could be carried out (Figure 1D). The rank clustering curve tends to flatten out, indicating that the amount of sequencing data is progressively reasonable (Figure 1C).

As can be seen from Table 2, the alpha diversity indices of groups A and B were mostly higher than those of group CK. The average number of visually observed OTUs, ACE index (estimation of the number of OTUs in the community), and PD_whole_tree index of each group showed CK < A < B (Figures 1E–G). The Shannon index of group A was higher than that of group CK (Figure 1H). The Simpson index did not differ significantly (*P* > 0.05) among groups (Figure 1J). In addition, ACE and Chao1 indices were significantly higher (*P* < 0.05) in group B than in groups CK and A (Figures 1F, K). The Goods_coverage index was significantly higher (*P* < 0.05) in group A than in groups CK and B (Figure 1I).

**Table 2:** Effects of prickly ash seeds (PAS) on microbial diversity of Hu lambs.

**Items**	**Group**			***P*-value**
	**CK**	**A**	**B**	
Observed_otus	1,808 ± 156.02^b^	1,911 ± 118.16	2,226 ± 201.16^a^	0.001
Shannon	8.25 ± 0.13	8.30 ± 0.12	8.70 ± 0.19	0.093
Simpson	0.99 ± 0.01	0.99 ± 0.01	1.00 ± 0.01	0.561
Chao1	2,103.37 ± 175.84^b^	2,254.24 ± 139.95^b^	2,607.26 ± 221.40^a^	0.001
ACE	2,151.46 ± 175.45^b^	2,298.81 ± 148.78^b^	2,659.21 ± 224.89^a^	0.001
Goods_coverage	0.991 ± 0.01^a^	0.989 ± 0.01^b^	0.988 ± 0.01^b^	0.001
PD_whole_tree	89.152 ± 5.69^b^	99.871 ± 11.88	112.66 ± 9.01^a^	0.002

CK, basal diet control; A, 3% PAS added group; B, 6% PAS added group. Means in the same row with different superscripts differ (*p* < 0.05).

### 3.2 Cluster analysis and microbial composition

**Figure 2:**
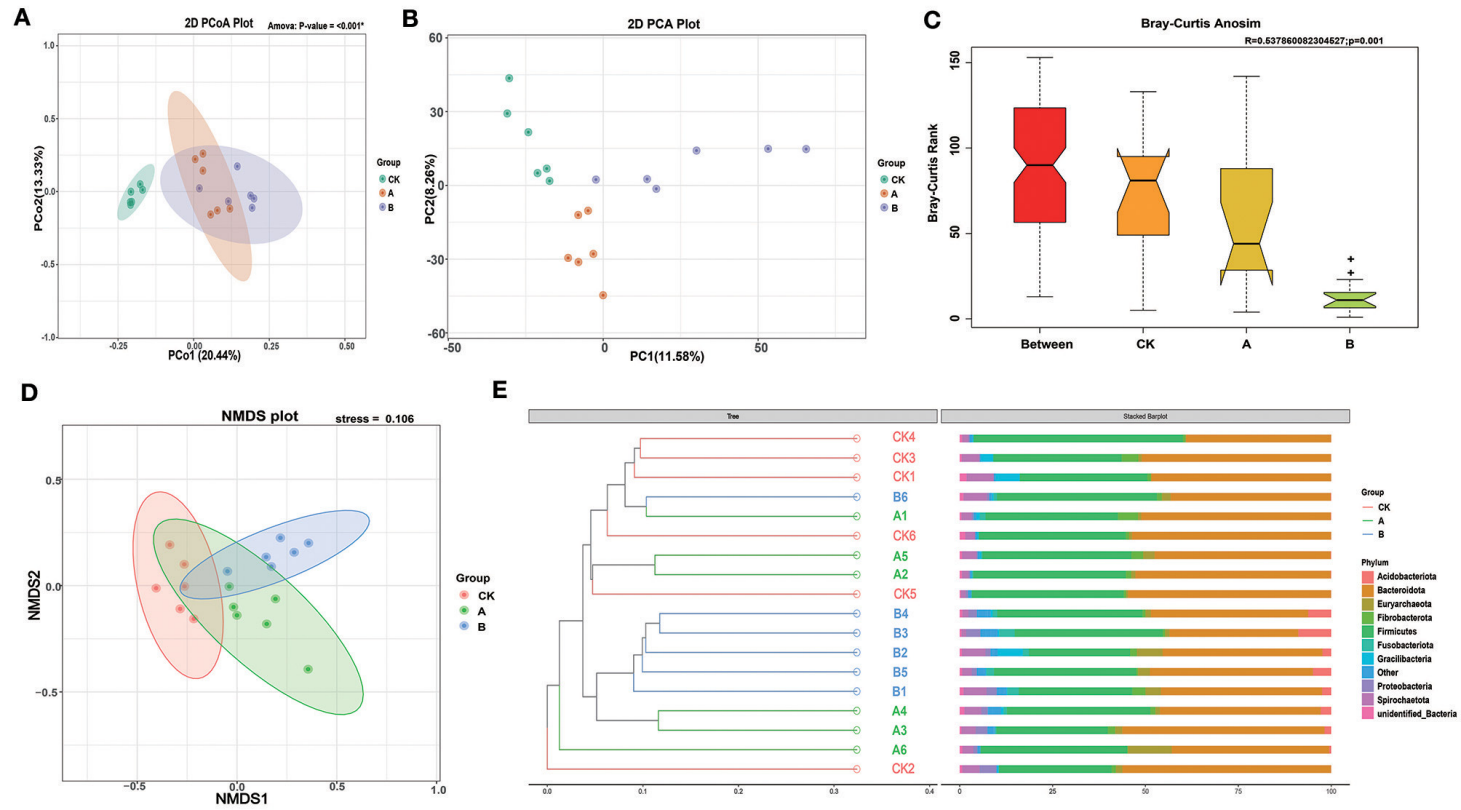
Cluster analysis and microbial composition. **(A)** Principal coordinate analysis (PCoA) based on all samples. **(B)** Principal component analysis (PCA) based on all samples. **(C)** OTU-based difference analysis of Anosim between groups. **(D)** Non-Metric Multi-Dimensional Scaling (NMDS) based on all samples. **(E)** Unweighted UniFrac distance UPGMA clustering tree based on OUT.

Principal coordinates analysis (PCoA) based on unweighted UniFrac showed that the rumen flora of lambs in group CK and groups A and B were significantly different, and the rumen flora of lambs in the two experimental groups were not significantly different (Figure 2A). PCA and NMDS analysis showed that the community composition was relatively similar within groups, but the community composition was more different between groups (Figures 2B, D). Moreover, the stress < 2 in NMDS analysis indicated that NMDS could accurately reflect the degree of differences between samples. The PCA plot showed that the contribution values of the two principal components were 11.58% and 8.26%, respectively, and group B and group A could be distinguished from the other two groups according to PC1 and PC2, respectively. ANOSIM showed that the range of the R-value between CK, A, and B groups was [−1.1], and the *p*-value was 0.001, which indicated that the difference between groups was significantly greater than the difference within groups (*P* < 0.05) (Figure 2C). In addition, the Multi-Response Permutation Procedure (MRPP) analysis and the Adonis analysis also indicated that the reliability of this test was high (Table 3). The UPGMA clustering tree based on the unweighted UniFrac distances of OTUs shows that half of group A is clustered with group CK and the other half is clustered with group B. The main rumen bacterial groups were *Bacteroidota* and *Firmicutes*, and the composition of the bacterial groups was basically similar among the groups. Interestingly, *Acidobacteriota* was present alone in all individuals of group B and in some individuals of group A (Figure 2E).

**Table 3:** MRPP analysis and Adonis analysis based on Bray–Curtis distance.

	**MRPP**				**Adonis**			
**Group**	**A**	**Observed_delta**	**Expected_delta**	**Significance**	**MeanSqs**	**F.Model**	**R** ^2^	**Pr(**>**F)**
CK vs. A	0.06	0.53	0.56	0.002	0.356	2.518	0.201	0.003
CK vs. B	0.09	0.48	0.53	0.003	0.367	3.087	0.236	0.003
A vs. B	0.08	0.47	0.51	0.006	0.315	2.803	0.219	0.002

A, basal diet control; A, 3% PAS added group; B, 6% PAS added group.

### 3.3 Relative abundance of rumen flora at the phylum level

**Figure 3:**
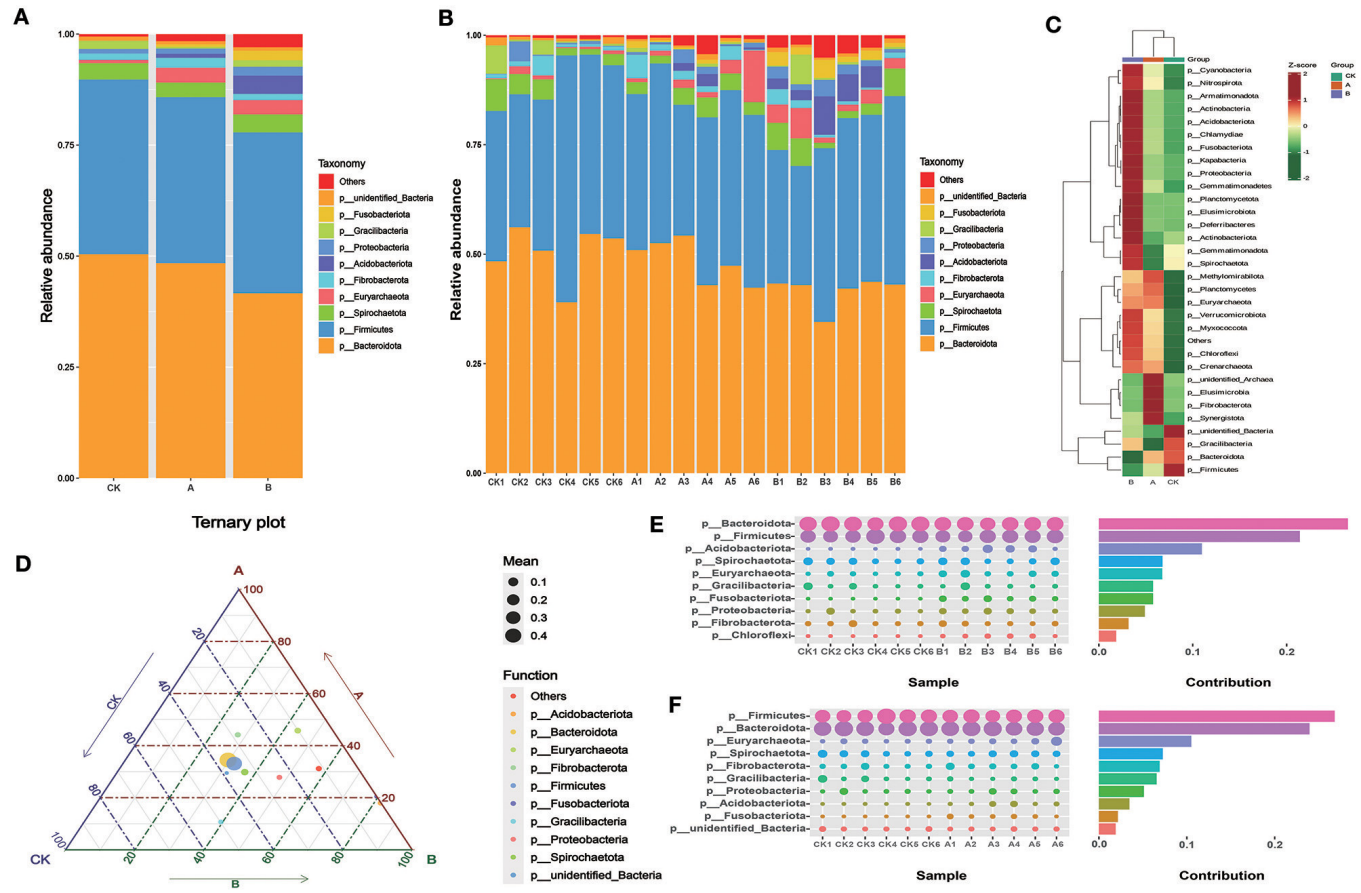
Relative abundance of rumen flora at the phylum level. **(A)** Microbial composition between the three groups at the phylum level. **(B)** Microbial composition of all samples at the phylum level between the three groups. **(C)** OTU-based species abundance heat maps for different groups at the phylum level. **(D)** Ternary-phase diagram based on the phylum level. **(E)** Contribution of Simper difference between group CK and group A based on the phylum level. **(F)** Contribution of Simper difference between group CK and group B based on the phylum level.

As shown in Table 4 and Figure 3A, *Bacteroidota* and *Firmicutes* were the main bacterial phyla comprising the rumen fluid flora of sheep lambs in the three groups, and their relative abundance accounted for more than 77.85% of the proportion of rumen flora. The abundance of *Fusobacteriota, Proteobacteria, Acidobacteriota*, and *Euryarchaeota* was lower in the CK group than in groups A and B. *Gracilibacteria* abundance was higher in the CK group than in groups A and B. The abundance of *Fibrobacteriota* and *Euryarchaeota* was higher in group A than in groups CK and B. The structure of the bacterial flora at the phylum level was basically similar for all samples within each group (Figure 3B). As can be seen from the heat map, most of the flora are higher in group B than in the other two groups in terms of content (Figure 3C). From the ternary plot, it can be seen that the abundance of *Gracilibacteria* was highest in group CK and *Acidobacteriota, Proteobacteria*, and *Firmicutes* in group B (Figure 3D). The final analysis of the species that differed between groups using SIMPLE revealed similar results to those of the previous graphs (Figures 3E, F).

**Table 4:** Relative abundance of species at phylum and genus levels.

**Items**	**CK Group**	**A Group**	**B Group**	***P*-value**
Phylum				
Bacteroidota	50.41^a^	48.39^a^	41.61^b^	0.021
Euryarchaeota	0.74	3.34	3.22	0.214
Fibrobacterota	1.44	2.26	1.41	0.559
Firmicutes	39.35	37.39	36.24	0.730
Gracilibacteria	1.84	0.39	1.46	0.492
Proteobacteria	1.03	1.17	2.01	0.420
Spirochaetota	3.67	3.28	4.04	0.810
Fusobacteriota	0.00^b^	0.56^ab^	2.16^a^	0.001
Acidobacteriota	0.01^b^	0.90^ab^	4.11^a^	0.005
unidentified_Bacteria	0.93	0.70	0.76	0.629
Others	0.59	1.62	2.98	0.014
Genus				
Papillibacter	0.66^b^	1.70^a^	1.25^a^	0.001
unidentified_Gracilibacteria	1.84	0.39	1.46	0.492
Bacteroides	3.45	0.22	0.65	0.182
Fibrobacter	1.44	2.25	1.41	0.562
Ruminococcus	2.56^a^	2.25^a^	1.27^b^	0.017
Methanobrevibacter	0.73	3.29	3.20	0.211
Saccharofermentans	2.47^b^	3.50^b^	4.23^a^	0.027
Succiniclasticum	4.32^a^	3.62^a^	1.47^b^	0.025
Treponema	3.63	3.20	4.00	0.791
Prevotella	15.25^a^	15.23^a^	10.22^b^	0.046
Others	63.65	64.40	68.35	0.368

CK, basal diet control; A, 3% PAS added group; B, 6% PAS added group. Means in the same row with different superscripts differ (*p* < 0.05).

### 3.4 Relative abundance of rumen flora at genus level

**Figure 4:**
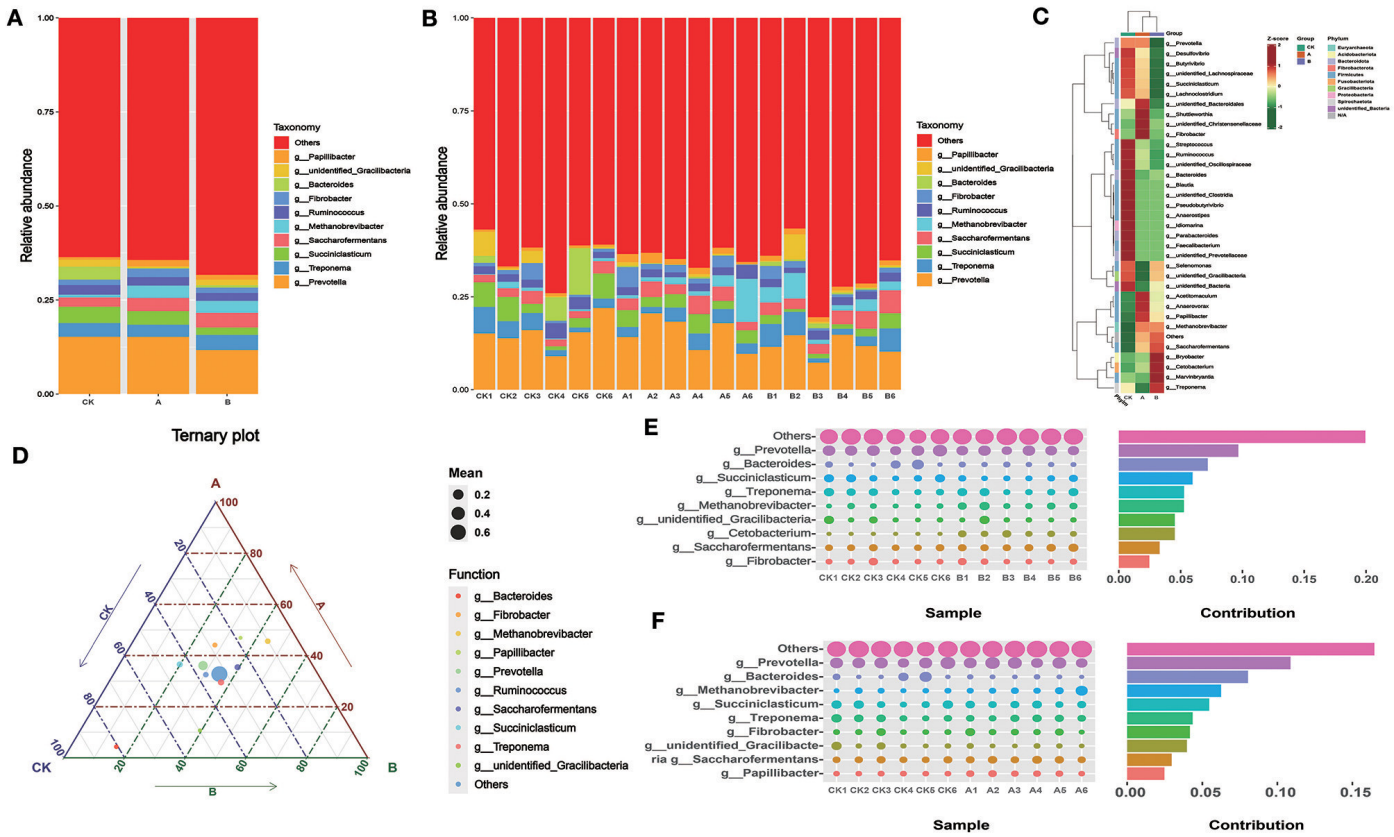
Relative abundance of rumen flora at the genus level. **(A)** Microbial composition between the three groups at the genus level. **(B)** Microbial composition of all samples at the genus level between the three groups **(C)** OTU-based species abundance heat maps for different groups at the genus level. **(D)** Ternary-phase diagram based on the genus level. **(E)** Contribution of Simper difference between group CK and group A based on the genus level. **(F)** Contribution of Simper difference between group CK and group B based on the genus level.

According to Table 4 and Figure 4A, at the genus level, the abundance of *Papillibacter* in the CK group was significantly lower than that in groups A and B (*P* < 0.05). The abundances of *Bacteroides, Ruminococcus, Succiniclasticum*, and *Prevotella* in group CK were higher than those in groups A and B. The abundance of *Saccharofermentans* in group B was higher than that in groups CK and A (*P* < 0.05). There was no significant difference in the abundance of other bacteria between the control group and the experimental group. There was no significant difference in the microflora content among all groups (Figure 4B). According to the heat map, compared with other groups, the bacteria in group CK with different expression content primarily belong to *Firmicutes* (Figure 4C). The content of *Bacteroides* in group CK was higher than that in groups A and B (Figures 4D–F). The content of *Methanobrevibacter* in groups A and B was higher than that in group CK (Figures 4E, F).

### 3.5 LEFse analysis and function prediction

**Figure 5:**
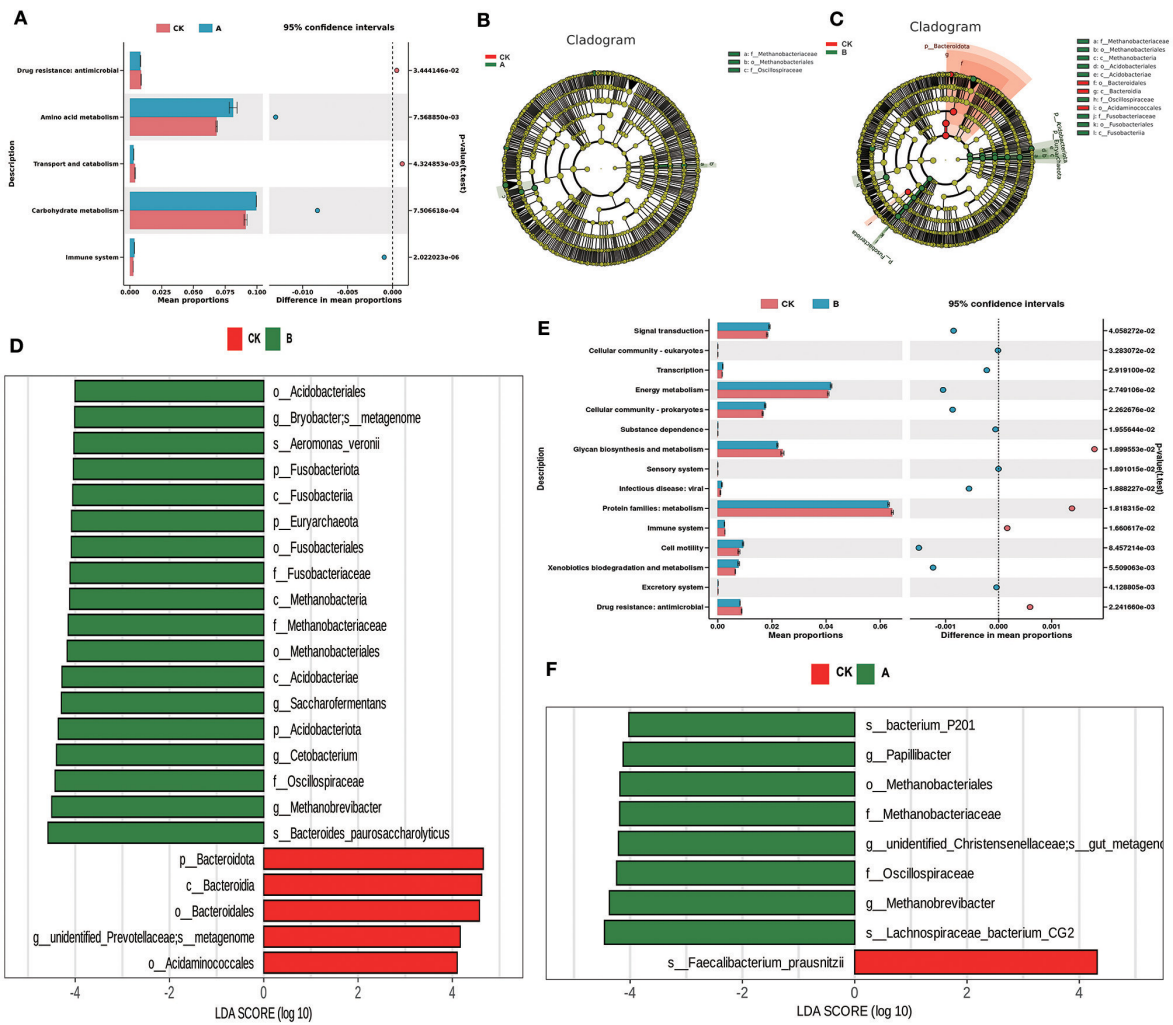
LEfSe analysis and PICRUST2 function prediction. **(A)** Functional differences between group CK and group A. **(B)** OTU-based evolutionary branching diagram between group CK and group A. **(C)** OTU-based evolutionary branching diagram between group CK and group B. **(D)** LDA value distribution histogram based on OTU between group CK and group B. **(E)** Functional differences between group CK and group B. **(F)** LDA value distribution histogram based on OTU between group CK and group A.

According to LEFse analysis, significant differences occurred in the dominant flora of prickly ash seeds added in the rumen. A comparison of group CK and group A revealed that the dominant flora of the CK group was *Faecalibacterium_prausnitzii*. The dominant flora of group A was *bacterium_P201, Papillibacteria, Oscillospiraceae, Methanobacteriales*, and *Lachnospiraceae_bacterium_CG2* (Figures 5B, F). A comparison of group CK and group B revealed that the dominant flora of group CK were *Bactreoidota, unidentified_Prevotellaceae*, and *Acidaminococcales*. The dominant flora of group B are then *Fusobacteriota, Euryarchaeota, Bryobacter, Aeromonas_veronii, Fusobacteriaceae, Acidobacteriota, Saccharofermentans*, Oscillospiraceae, *Cetobacterium*, and *Bacteroides_paurosaccharolyticus* (Figures 5C, D). The PICRUSt2 software package was used for functional prediction based on the KEGG database based on 16s sequencing data, and Stamp maps were drawn. The results showed that amino acid metabolism, carbohydrate metabolism, and the immune system in group A were significantly higher than those in group CK (*P* < 0.05), and antimicrobial activity and transport and catabolism in group CK were significantly higher than those in group A (*P* < 0.05) (Figure 5A). After comparison between group CK and group B, it was found that signal transduction, transcription, energy metabolism, cellular community-prokaryotes, cell motility and xenobiotics biodegradation, and metabolism in group B were significantly higher than those of group CK (*P* < 0.05). Glycan biosynthesis and metabolism, immune system, and antimicrobial activity in group CK were significantly higher than those in group B (Figure 5E).

### 3.6 Correlation analysis of rumen microorganism and production traits

**Figure 6:**
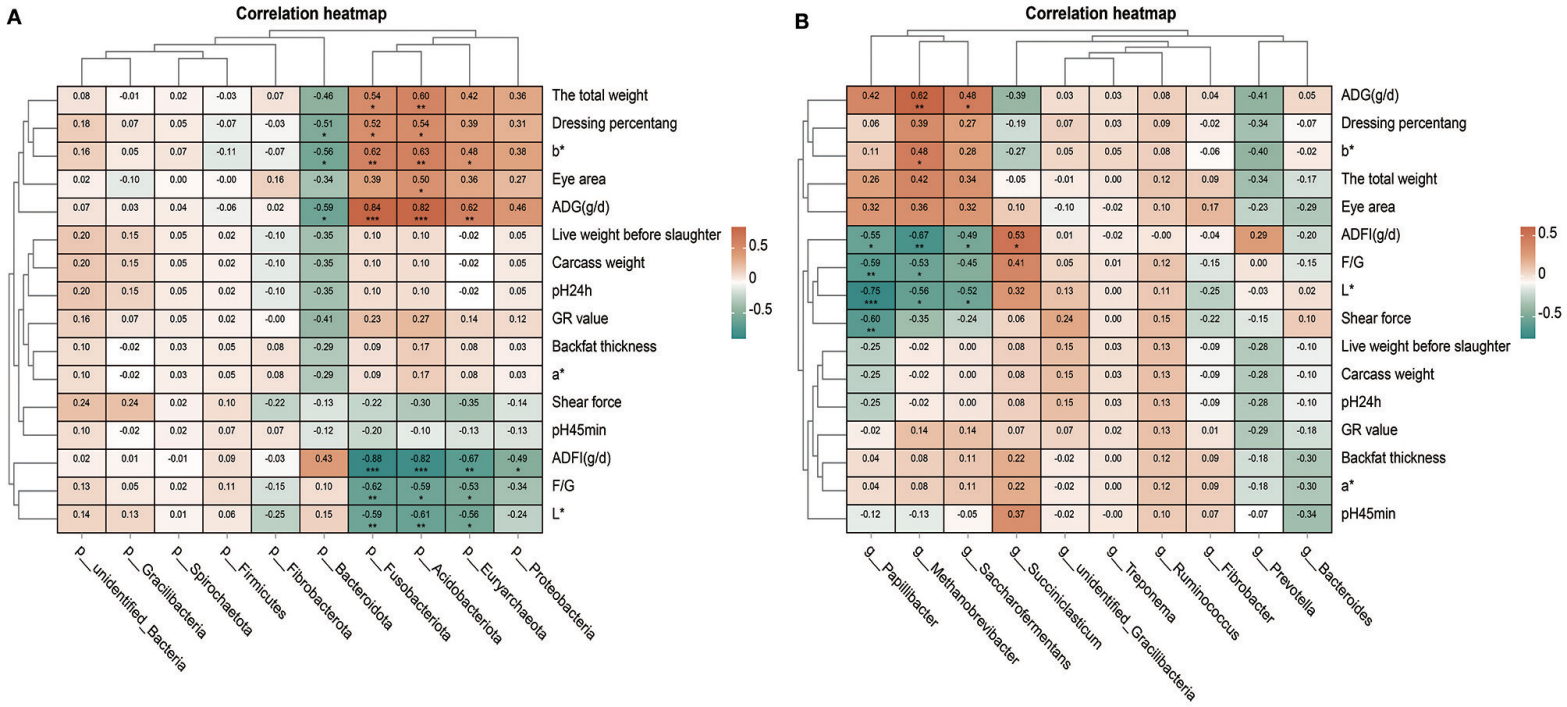
Correlation analysis between microorganisms and production traits. **(A)** Thermal map analysis of correlation between phylum level microorganisms and production traits. **(B)** Thermal map analysis of correlation between genus level microorganisms and production traits.

Through the analysis of the correlation between the phylum or genus level microorganisms and production traits, we found that many microorganisms had a significant correlation with production traits (Figures 6A, B). We have found many interesting phenomena. For example, b^*^ and ADG(g/d) are positively correlated with *Fusobacteriota, Acidobacteriota, Euryarchaeota*, and *Methanobrevibacter* and negatively correlated with *Bacteroidota*. The total weight and dressing percentage were positively correlated with *Fusobacteriota* and *Acidobacteriota*. ADFI(g/d), F/G(g/d), and L^*^ are negatively correlated with *Fusobacteriota, Acidobacteriota, Methanobrevibacter*, and *Papillibacter*. In addition, dressing percentage was negatively correlated with *Bacteroidota*. ADFI(g/d) was negatively correlated with *Proteobacteria* but positively correlated with *Succiniclasticum*. *Saccharofermentans* was positively correlated with ADG(g/d) but negatively correlated with ADFI(g/d) and L^*^. Shear force was negatively correlated with *Papillibacter*.

## 4 Discussion

As an important digestive organ of ruminants, the rumen microbes cannot function without the presence of various microorganisms in it, and the microbial diversity is closely related to species, age, diet, and feeding environment (Loh et al., 2020; Gresner et al., 2022). The digestion of more than 70% of carbohydrates and proteins in the feed is completed by rumen microorganisms (Wang et al., 2020; Zhang et al., 2021). Therefore, diet composition is an important factor affecting the structure and function of rumen microflora (Chai et al., 2021; Ge et al., 2023). Alpha diversity indexes can reflect the richness and diversity of species in the samples, of which the ACE index and Chao1 index were mainly related to the richness of microbial communities, and the larger the value, which indicates that there were more species in the samples, the higher the richness; whereas the Shannon index and the Simpson index were related to the diversity of microbial communities; the larger the Shannon index, the higher the diversity of microbial communities, and the smaller the Simpson index, the higher the diversity of microbial communities (Jiang et al., 2022; Li et al., 2022). Rumen microorganisms play an important role in the health and growth of sheep, and the higher the diversity of rumen microorganisms, the more stable the microbial ecosystem, which is more beneficial to the growth of sheep (Wang et al., 2023b). In this experiment, through alpha diversity index analysis, it was found that the Chao1 and Ace indices of the two experimental groups were significantly higher than those of the control group, indicating that prickly ash seeds had a significant impact on the abundance of rumen microorganisms, and rumen flora was more abundant in the experimental group. The beta diversity index is a comparative analysis of the microbial community composition of different samples. We choose four methods to carry out this analysis. All the methods showed that the degree of microbial community differences within groups was smaller than that between groups. PCoA showed that the CK group and two experimental groups were significantly separated. PCA and NMDS analysis showed that the three groups could be distinguished from each other. ANOSIM showed that there were significant differences among all groups. However, the three groups of sheep were not significantly separated in NMDS analysis, which was significantly different from PCA and PCoA, which may be due to differences in the clustering methods. At the same time, through the UPGMA tree diagram at the phylum level, we found that the dominant bacteria genera of all the sheep tested at the phylum level were *Bacteroidota* and *Firmicutes*, and the rumen flora structure in group CK and group B was similar, while the rumen flora structure in group A was different. Although the rumen microflora structure of sheep was affected by the amount of Prickly Ash Seeds (PAS) added, there were still individual differences in the same group of sheep (Sato et al., 2023).

There is a symbiotic relationship between ruminants and rumen microorganisms. The organism provides a suitable environment for microorganisms to grow, while the microorganisms decompose nutrients in the rumen for absorption and utilization by the organism (Malmuthuge and Guan, 2017). The composition and difference of rumen microorganisms in three groups of sheep were analyzed. We found that, after the addition of PAS, the upregulated microorganisms in sheep rumen included *Methanobacteria, Papillibacter, Fusobacteriota, Acidobacteriota, Euryarchaeota, Saccharofermentans, Proteobacteria*, and *Oscillospiraceae*, among others. *Methanobacteria* is a group of specialized methane-producing microorganisms that exist in the rumen of ruminants. They convert hydrogen and carbon dioxide or carbon monoxide into methane via the methanation pathway, a process that is critical for pH balance and energy metabolism in the rumen. In the gut of animals such as sheep (Drancourt et al., 2021), *Euryarchaeota* is also involved in methane production (Goffredi et al., 2008). *Papillibacter* helps the host break down food, promotes nutrient absorption, and is involved in maintaining microecological balance in the gut (Bonyadian et al., 2018). *Saccharofermentans* can produce acetic acid by fermenting carbohydrates, which contributes to the acidification of the rumen environment and the energy supply of the animals. *Oscillospiraceae* helps the host digest, absorb nutrients, and maintain intestinal barrier function (Aljumaah et al., 2022). In addition, it was found that the microorganisms that decreased the rumen content after PAS addition included *Bacteroidota, Acidaminococcales, Faecalibacterium_Prausnitzii, Firmicutes, Gracilibacteria, Bacteroides, Prevotella*, and *Ruminococcus*. *Ruminococcus* plays an important role in digesting resistant starch, and *Prevotella* is often considered a bacterium associated with a healthy plant-based diet, acting as a “probiotic” in the human body (Cheng et al., 2023). *Prevotella* plays a role in the diagnosis and treatment of inflammation-related diseases (Yang et al., 2018). *Firmicutes* can indirectly connect with other tissues and organs through metabolites and regulate hunger and satiety (Zhou et al., 2022a). *Bacteroidetes* and *Firmicutes* are often the “core microbiome” of rumen in ruminants, and this study was no exception in substantiating the abovementioned finding (Weimer, 2015). These two dominant phyla work together to promote the degradation of fibrous and non-fibrous materials in the rumen to produce acetic acid and propionic acid for absorption and utilization by the body (Ye et al., 2022; Zhou et al., 2022b). As a dominant phylum in the rumen, the main role of *Bacteroidota* is to promote the degradation of complex organic matter in ruminants, such as the degradation of carbohydrates, which can help the host to absorb and utilize polysaccharides efficiently (An et al., 2023; Wang et al., 2023c). Therefore, the experiment showed that the high addition of prickly ash seeds may have negative effects on the digestive function in the rumen. In conclusion, the richness and diversity of rumen flora of sheep can be enhanced by adding prickly ash seeds in the diet, and the diet supplemented with 3% prickly ash seeds may be more beneficial to the healthy development of sheep rumen. Some beneficial bacteria that have special effects in improving immunity and other aspects may work together to enhance sheep production traits.

Based on the above research results and the previous research results (Ma et al., 2024), the correlation analysis was carried out on the relationship between rumen microorganisms and production traits. It was found that *Papillibacter* were inversely correlated to ADFI(g/d), F/G, L^*^, and shear force, and *Papillibacter* may be associated with weight management and obesity (Zong et al., 2023). *Saccharofermentans* belongs to butyric acid-oxidizing bacteria, which can produce short-chain fatty acids such as butyric acid through fermentation (Li et al., 2018). With the addition of PAS, *Saccharofermentans* content and ADG(g/d) increased. It is likely that *Saccharofermentans* can reduce ADFI(g/d) while increasing ADG(g/d) in sheep. *Methanobrevibacter* is positively correlated with ADG(g/d) and may play a role in weight regulation by affecting the digestion of food and the absorption of calories. *Euryarchaeota* is able to produce a short-chain fatty acid called butyric acid, which is believed to help control appetite and fat storage (He et al., 2022; Li et al., 2023b). *Fusobacteriota* can also produce metabolites that are beneficial to the human body, such as pyruvate and butyric acid, which play an important role in intestinal health and the regulation of the immune system (Bleicher et al., 2022; Guo et al., 2022). It was found that *Fusobacteriota, Acidobacteriota*, and *Euryarchaeota* were positively correlated with ADG and negatively correlated with ADFI and F/G. The increase of these bacteria content may contribute to the increase of the average daily gain of sheep, the decrease of the average daily feed intake, and the decrease of the feed to gain ratio.

## 5 Conclusion

This study revealed the effects of adding different proportions of prickly ash seeds on the rumen flora of Hu sheep lambs and correlated the top ten bacteria in terms of relative abundance of species at the phylum and genus level with production traits. The results showed that the addition of different levels of prickly ash seeds could increase the diversity and abundance of sheep rumen microorganisms, influence the composition of sheep rumen community, and increase the content of rumen beneficial bacteria. It also promoted metabolic capacity and immune system in lambs. However, the addition of 6% prickly ash seeds resulted in greater changes in rumen flora, an increase in harmful bacteria, and a possible decrease in the excretory system and the immune system. Correlation analysis showed that *Fusobacteriota, Acidobacteriota, Euryarchaeota*, and *Methanobrevibacter* were positively correlated with ADG(g/d) and negatively correlated with ADFI(g/d) and F/G. Therefore, the present study provides new insights on improving rumen health of sheep through the addition of green additives and provides some help in understanding the correlation between rumen flora and production traits.

## Data availability statement

The datasets presented in this study can be found in online repositories. The names of the repository/repositories and accession number(s) can be found below: https://www.ncbi.nlm.nih.gov/, PRJNA1058965.

## Ethics statement

The animal studies were approved by the Animal Care Committee of Gansu Agricultural University (GSAU-AEW-2020-0057). The studies were conducted in accordance with the local legislation and institutional requirements. Written informed consent was obtained from the owners for the participation of their animals in this study. The studies were conducted in accordance with the local legislation and institutional requirements. Written informed consent was obtained from the owners for the participation of their animals in this study.

## Author contributions

DL: Methodology, Writing – original draft, Writing – review & editing. QL: Writing – review & editing. XM: Writing – review & editing. HuW: Writing – review & editing. CW: Writing – review & editing. HaW: Writing – review & editing. ZL: Writing – review & editing. TL: Writing – original draft, Writing – review & editing. YM: Methodology, Writing – original draft, Writing – review & editing.
