# Supplementary material for: Prickly ash seeds can promote healthy production of sheep by regulating the rumen microbial community
Source: Front Microbiol. 2024 May 20;15:1364517. doi: 10.3389/fmicb.2024.1364517 (PMC11144891; doi:10.3389/fmicb.2024.1364517)
Supplement: Supplementary file 1 [file Table_1.DOCX]

Supplementary table1 Content of conventional nutrients in Prickly ash seeds

| Items | Content/% | Items | Content/% |
| --- | --- | --- | --- |
| Crude fat | 22.50 | ADF | 26.35 |
| Crude protein | 15.60 | Ca | 0.26 |
| NDF | 40.23 | P | 0.47 |
| Dry matter/% | 90.60 |  |  |
